# What moral weight should patient‐led demand have in clinical decisions about assisted reproductive technologies?

**DOI:** 10.1111/bioe.13239

**Published:** 2023-11-06

**Authors:** Craig Stanbury, Wendy Lipworth, Siun Gallagher, Robert J. Norman, Ainsley J. Newson

**Affiliations:** ^1^ Department of Philosophy, Monash Bioethics Centre Monash University Clayton Victoria Australia; ^2^ Sydney Health Ethics, Sydney School of Public Health, Faculty of Medicine and Health The University of Sydney Sydney New South Wales Australia; ^3^ Department of Philosophy, Macquarie University Research Centre for Agency, Values and Ethics Macquarie University Macquarie Park New South Wales Australia; ^4^ Robinson Research Institute Adelaide University Adelaide South Australia Australia

**Keywords:** assisted reproductive technologies, autonomy, commercialisation, harm, patient demand, professional role morality

## Abstract

Evidence suggests that one reason doctors provide certain interventions in assisted reproductive technologies (ART) is because of patient demand. This is particularly the case when it comes to unproven interventions such as ‘add‐ons’ to in vitro fertilisation (IVF) cycles, or providing IVF cycles that are highly unlikely to succeed. Doctors tend to accede to demands for such interventions because patients are willing to do and pay ‘whatever it takes’ to have a baby. However, there is uncertainty as to what moral weight should be placed on patient‐led demands in ART, including whether it is acceptable for such demands to be invoked as a justification for intervention. We address this issue in this paper. We start by elucidating what we mean by ‘patient‐led demand’ and synthesise some of the evidence for this phenomenon. We then argue that a doctor's professional role morality (PRM) yields special responsibilities, particularly in commercialised healthcare settings such as ART, because of the nature of professions as social institutions that are distinct from markets. We argue on this basis that, while there may be reasons (consistent with PRM) for doctors to accede to patient demand, this is not always the case. There is often a gap in justification between acceding to patient‐led demands and providing contested interventions, particularly in commercial settings. As a result, acceding to demand in such settings needs a strong justification to be consistent with PRM.

## 1 INTRODUCTION

Assisted reproductive technologies (ART) are a group of interventions used with the aim of establishing pregnancies in individuals who require assistance with conception. They mainly comprise in vitro fertilisation (IVF), but also include intrauterine insemination, donor insemination and other procedures. Each year, around 2.5 million IVF treatment cycles are carried out globally, resulting in around 0.5 million births.^1^ IVF, however, is expensive, can be physically and psychologically onerous and is frequently unsuccessful.^2^ Deciding to commence IVF gives rise to many choices for doctors and patients, including whether and when to use treatment, how many cycles to attempt and whether to supplement standard treatment protocols with additional interventions, referred to as ‘add‐ons’. Such choices can be contentious. For example, many add‐ons are not yet supported by sufficient evidence to be considered part of ‘standard practice’ and may be costly and harmful.^3^ Additionally, some observers of the ART industry have raised concerns about the premature commencement of IVF and continuation of IVF cycles when success is unlikely.^4^

One justification sometimes given for offering interventions in ART is that patients request or even demand them. Some might argue that, when faced with such requests and demands, doctors should simply accede because patients are consumers whose desires for goods and services should be fulfilled, as in any other commercial transaction. Others, in contrast, might argue that doctors should assume a position of benevolent paternalism in which the ‘doctor decides’. One alternative to this dichotomy is ‘shared decision‐making’, where doctors and patients work together to define outcomes consistent with the patient's wishes and goals.^5^ This approach, however, raises its own dilemmas—for example, how to prevent shared decision‐making from devolving into disguised consumerism or paternalism, and how to account for the fact that patients vary in the degree to which they want, and are able, to participate actively in decision‐making.^6^ Most importantly, for the purposes of this paper, shared decision‐making does not provide a clear answer as to what to do when doctors and patients cannot reach a satisfactory shared decision—that is, when a patient requests or demands something that a doctor does not wish to offer. In such circumstances, we need to determine how much moral weight should be placed on patient‐led demands.

In this paper, we argue that professional role morality (PRM) can help address questions about how to respond to patient requests and demands in ART. In Section 2, we offer a working definition of ‘patient‐led demand’ and synthesise evidence of this phenomenon in various settings, including ART. In Section 3, we investigate what a doctor's PRM amounts to and how it yields certain responsibilities and obligations. In Section 4, we apply PRM to patient‐led demand in ART. We argue that, while there might be some circumstances in which patient‐led demand carries enough moral weight to justify an intervention, this is not always the case. In Section 5, we argue that commercial influences both add further complexity to the question of when and how to respond to patient requests and demands, and create further obligations for doctors who accede to such demands. We conclude that there is a gap in justification between acceding to patient‐led demands and providing contested interventions, particularly in commercial settings, and that, in these settings, acceding to patient‐led demand needs strong moral justification in order to be consistent with professional role obligations.

## 2 PATIENT‐LED DEMANDS FOR HEALTHCARE INTERVENTIONS

The growth of the Internet and widespread use of social media have increased the phenomenon of patients approaching their doctors with requests for interventions that they believe will help them.^7^ If the doctor and patient agree that the requested intervention is justified on grounds such as it being in the best interests of the patient, then patient requests are prima facie morally unproblematic.^8^ Sometimes, however, a patient's request might conflict with what a doctor believes is in the patient's best interest. This request may persist even after the doctor has provided an explanation, with the patient stating or intimating that they will consider themselves wronged in some way if they do not receive the intervention that they desire. We refer to this phenomenon as ‘patient‐led demand’.^9^ When a patient's demand is not satisfied, they might reluctantly accede to the doctor's advice, transfer their care to another doctor or eschew care altogether.

Patient‐led demand has been reported in several areas of medicine, including in obstetrics,^10^ oncology,^11^ antibiotic prescribing,^12^ novel pharmaceuticals,^13^ aesthetic medicine and dentistry^14^ and ART.^15^ The results of a study that we conducted into ART in Australia also suggest that patient‐led demand occurs in this context.^16^ We found that doctors accede to patient‐led demand for various reasons, including viewing patients as consumers, not wanting to lose patients to a competitor provider or believing that saying ‘no’ will cause patients psychological harm.

While some of our participants were unconcerned by doctors acceding to patient‐led demand—particularly if no harm was likely, or if they emphasised patients as empowered consumers—others reported being troubled by their own or others' practice of acceding to patient‐led demand. This kind of disagreement and ambivalence is not surprising, given the complexities of autonomy, benefit and harm in the ART context. Some might argue that autonomy should always take precedence, even if there is considerable risk involved. Others might counter that concepts such as beneficence or nonmaleficence should always take precedence over autonomy, either because autonomy is not as important as these other notions, or because preferences are not always reached autonomously, even in patients who are themselves autonomous.^17^ The latter consideration is particularly salient in the ART context because societal pressures to have a baby and the significant hope invested in procreation can influence preferences without patients (or providers) realising or appreciating this. This approach may, however, be challenged on the grounds that even patients experiencing social and psychological pressure retain a sufficient degree of autonomy for this to warrant respect, or on the grounds that a patient's best interests might still be upheld even when they are making a cognitively biased decision.^18^ As such, appeals to principles such as autonomy and beneficence cannot, on their own, solve the practical quandary that doctors face when patients demand interventions that the doctor believes are not in the patient's interests.

There are several potential ways to approach the ethical challenge posed by patient‐led demand. One could attempt to determine (theoretically and/or empirically) what constitutes a desirable outcome and then conduct empirical research to determine whether acceding to patient‐led demand ultimately results in better outcomes than resisting such demands. One could reason from cases or narratives to consider how a ‘virtuous’ clinician would act in relation to patient demand. Or one could reflect on a doctor's PRM, asking what specific duties doctors have *qua* being doctors. In what follows, we explore this last perspective.

## 3 PROFFESSIONAL ROLE MORALITY

Some moral obligations apply universally: unjustified killing, corruption and oppression are wrong regardless of who you are. However, other moral obligations apply only to people who occupy particular roles.^19^ Parents, for example, are expected to be partial to and care for their children in a way others are not. ‘Role morality’ refers to the responsibilities and obligations that someone has simply because of the role that they occupy. Chappell explains:

A role is a moral, legal, political, institutional, or social persona or function or office or guise or *qua* … that brings with it distinctive responsibilities, privileges, powers, immunities, and expectations of the relevant kind—moral, legal, political, or whatever.^20^

Roles can take a variety of forms. Parents, for instance, hold a *social* role that forms constraints and duties toward their children. Doctors, on the other hand, hold a *professional* role. Professional roles—as opposed to nonprofessional occupations—are generally distinguished by three criteria.^21^ First, they must serve an essential human need rather than merely fulfil desires. The roles held by doctors can be said to meet this criterion because health is essential to human flourishing.^22^ Second, professional roles involve specialist training that enables autonomy and professional judgement to be exercised within the role. According to Wolfendale:

Professionals are not mere technicians for hire or automata carrying out rote tasks—their roles require judgement, reflection, and wisdom. Professionals are reflective moral agents who must make informed decisions about when and how to apply their professional knowledge, decisions involving significant responsibility for their client's wellbeing and interests.^23^

The highly specialised nature of professional roles means that those who occupy them hold a monopoly on the valued goods provided.^24^ They are granted the authority and resources to carry out their roles, and a considerable degree of autonomy in setting and overseeing professional standards.^25^

Because of their monopoly on values central to human flourishing, and their privilege and autonomy, the third criterion of a professional role is that professionals must acknowledge the moral dimension inherent to their role. Once someone is a professional, recognises that they occupy this role and acknowledges that the responsibilities and duties of the role apply to them, professional obligations form parameters around their conduct^26^ (e.g., precluding them from being driven solely by financial rewards)^27^ and give them strong *moral* reasons for acting in particular ways.

What counts as acting well in the context of a *specific* professional role can be reasonably determined by how well the action serves the *goals* of the profession. Oakley and Cocking elaborate this point in relation to the medical profession:

…an appropriately action‐guiding professional ethic cannot be generated without specifying what the appropriate orientation and essential guiding concerns of the particular profession ought to be. Thus, the content of the regulative ideals of a good doctor must be determined by reference to some model of what doctoring purports to be. That is, those regulative ideals will be informed by an account of the proper goals of medicine as a practice.^28^

A *good* doctor, then, is someone who attempts to achieve the goals of their profession. And they are likely doing something *wrong* if they act contrary to the goals of their role without a clear justification for doing so.^29^

There is currently broad acceptance both within and outside the profession that the goals of being a doctor include enhancing patients' health and well‐being, relieving suffering and promoting human flourishing. Miller and Brody, for example, explain that medical practice can be encompassed by three goals: healing, promoting health and helping patients achieve a dignified death.^30^ Similarly, The Hastings Center identifies the four goals of medicine as (1) The prevention of disease and injury and the promotion and maintenance of health; (2) The relief of pain and suffering caused by maladies; (3) The care and cure of those with a malady, and the care of those who cannot be cured; and (4) The avoidance of premature death and the promotion of a peaceful death.^31^

Rhodes synthesises various professional obligations that follow from the goals of the profession and that have a broad consensus in medical codes of ethics.^32^ They include to:

- Seek (patient and society) trust and be deserving of it (i.e., to be ‘trustworthy’);
- Promote the best health interests of patients and society, rather than act in their own (financial or nonfinancial) interests;
- Commit to science‐based (evidence‐based) practice;
- Respect the patient's autonomy within the bounds of what is in the patient's interests and consistent with accepted standards of care;
- Uphold the standards and reputation of the profession; and
- Practice medicine competently.

Further, because pursuing the goals of medicine can result in harmful side effects (such as those caused by chemotherapy when treating cancer), doctors have a duty to avoid harm that is ‘not balanced by the prospect of compensating medical benefits’.^33^

In the next section, we apply this thinking to the issue of patient‐led demand in ART. Before doing so, we acknowledge that PRM is not the *only* guide to right action in medicine. We also acknowledge that it can be morally problematic at times to conflate PRM with ethics because groups of professionals sometimes advocate—on the basis of their own ‘morality’—for policies that benefit them rather than those they are meant to serve, including using their accounts of professionalism to defend themselves against external critique.^34^ Another limitation of PRM is that it tends to focus on professional attributes such as virtues and duties that may be insufficient to guide ethical behaviour in all situations or poorly suited to addressing issues that do not attach to a particular role.^35^

That said, PRM is an integral part of the tapestry of professional morality, as evident in the prominence given to professionalism in medical training.^36^ There is also the long history^37^ and ongoing development of professional codes of ethics and practice in medical professional organisations and their invocation in professional disciplinary proceedings when legal standards are absent, unclear or fail to articulate morally important dimensions of practice.^38^ For example, the American Board of Internal Medicine (ABIM) Physician Charter, which has over 130 signatory organisations, views the medical profession as having entered into a social contract with society, so it demands particular priorities and standards of behaviour^39^:

Professionalism is the basis of medicine's contract with society. It demands placing the interests of patients above those of the physician, setting and maintaining standards of competence and integrity, and providing expert advice to society on matters of health. The principles and responsibilities of medical professionalism must be clearly understood by both the profession and society. Essential to this contract is public trust in physicians, which depends on the integrity of both individual physicians and the whole profession.

Because professional roles are *essential* to helping human lives flourish and because they are based on social contracts, professional obligations hold significant moral weight: acting *within* their role provides a professional with strong justification for their actions.^40^ Going against or not aligning with one's professional obligations undermines important agreements and human goods. It is, therefore, particularly important that professionals—arguably more so than occupations without professional status—uphold the obligations of their role, and while specific goals and obligations can be questioned and may not always trump other moral goods, acting contrary to them requires sound justification.

Scholars have applied PRM to debates about appropriate professional conduct in medicine in general^41^ and to issues such as managed care,^42^ the ethics of cosmetic surgery^43^ and conscientious objection in healthcare.^44^ Yet, to date, none have explored what PRM entails for ART provision in general or for patient‐led demand for ART interventions. In Section 4, we apply PRM to three clinical scenarios in ART and show that patient‐led demand can at times hold less moral weight than might be asserted or assumed.

## 4 APPLYING PROFESSIONAL ROLE MORALITY TO PATIENT‐LED DEMAND IN ART

To address the issue of whether (and, if so, when) patient‐led demand carries enough moral weight to justify treatment that a doctor would otherwise prefer not to provide in ART settings, we consider three clinical scenarios and explore how PRM might help to determine the right action in each. The scenarios have been developed to be both clinically realistic^45^ and to illustrate important considerations such as evidence standards and harms—both direct and indirect.^46^

***Scenario 1—Unclear clinical effects***: *A patient has had a poor response to hormonal stimulation on an IVF cycle and requests a prescription for dehydroepiandrosterone (DHEA), a natural steroid with no known side‐effects but of unproven value*.

In this scenario, evidence of benefit is unclear, but there is also little, if any, risk of direct physical harm. There are two main reasons why fulfilling this patient's request would be consistent with PRM (and why there is little likelihood that the request would ever need to devolve into a ‘demand’). First, respecting patient autonomy is an important part of a doctor's role obligations. Second, offering an intervention of uncertain benefit can be an example of clinical innovation, which is an accepted part of medical practice. It is common for doctors to use nonevidence‐based interventions because of the patient's unique circumstances or when patients have run out of therapeutic options. When this is done responsibly (e.g., with adequately informed consent, monitoring for harm, concurrent data collection and minimal or no cost to the patient), it is compatible with doctors' role morality.

Nevertheless, PRM also reminds doctors of the importance of also considering broader, indirect, harms and costs. Patients' hopes and finances can be particularly vulnerable to exploitation in ART settings. While DHEA might be inexpensive, other physically harmless add‐ons may cost hundreds or thousands of dollars extra per cycle. Even if a patient is willing to pay for this because they hope that it will benefit them, if the doctor believes that there is not enough evidence to reasonably believe that the add‐on will be successful, they risk causing *indirect* harm. PRM suggests that doctors must be sensitive to the economic, occupational and social conditions that impact a patient's health status.^47^ Doctors must also be sensitive to the indirect harms that can arise from hope that is not likely to be fulfilled.^48^ While doctors should not make unilateral decisions about what patients can afford financially, or handle psychologically, PRM suggests that they must engage with the patient sensitively about their demands and about competing moral (including psychological and financial) considerations. Where evidence of benefit and/or safety is unclear, PRM also demands that data be collected so that risks and costs borne by patients (whether or not they ‘demand’ interventions) contribute to shared knowledge about interventions.

***Scenario 2—Equivocal effects***: *A patient has had several unsuccessful IVF procedures and asks for an endometrial scratch technique. This involves outpatient insertion of a catheter into the uterus to scratch the uterine lining in the hope that this will trigger a local reaction that makes it more likely that an embryo will implant. Numerous randomised trials have shown lack of benefit, but this intervention is relatively inexpensive and not likely to cause physical harm apart from minor discomfort during the procedure*.^49^

This scenario, in which a patient demands an intervention *known not to* be effective, is more problematic from a PRM perspective. As in Scenario 1, acceding to patient‐led demand here is not necessarily inconsistent with PRM. But because there is not even a *chance* of direct physical benefit and clinical innovation cannot be invoked as a supporting justification, more moral work has to be done to ensure that there is *really* no likelihood of direct physical harm, that indirect harms and costs are given serious attention and that using the intervention is genuinely in line with the patient's preferences (e.g., that it is more important to them to ‘try everything’ than it is to avoid physical risk or financial cost).

An additional consideration here is that, as professionals, doctors have a special insight into their monopolised service. They are autonomous agents who are not mere automata or levers to be pulled for another's intentions. They have a role obligation to exercise this agency to apply their specialised knowledge to help the patient. If the evidence suggests that the intervention will not be successful, it seems even more important, then, that doctors consider any broader or indirect harms (such as exploiting a patient's hope or finances), as well as exploring in depth the patient's reasons for wanting the intervention to ensure that they are aware of the risks and benefits and are acting in a manner consistent with their values and preferences.

***Scenario 3—Evidently harmful effects***: *A young patient requests transfer of two high‐quality embryos rather than one. A slightly higher pregnancy rate will be obtained with two embryos, but sequential transfer (1 + 1) will give the same overall result. Two embryos will give rise to a 30% chance of a twin pregnancy and a 1% chance of triplets, with attendant increased risks of maternal morbidity and perinatal death. The clinic policy is, therefore, to only transfer one embryo at a time*.^50^

It is much more problematic from a PRM perspective to offer an intervention in this scenario, where the intervention that the patient is requesting is *not* beneficial and there is a significant likelihood of direct harm—that is, the benefit‐to‐harm ratio is not consistent with what would be considered good clinical practice. In this context, PRM would exclude offering the intervention *solely* because the patient demands it. ART doctors (explicitly or implicitly) have promised to consider and act on a range of reasons consistent with the role morality of their position. This includes an understanding not to perform actions or to act on reasons that do not fit the proper goals of their role^51^ unless there is a strong moral justification for doing so. While some patients might want multiple embryos transferred because of their age or to avoid the costs of additional IVF cycles, there is broad professional agreement that the risks to patients and costs to health systems are so great that such desires should not be accommodated. In this scenario, acceding to patient‐led demand is clearly inconsistent with both the goals of medicine—to promote the best interests of a patient's health—and the corresponding obligations of doctors to commit to evidence‐based practice and avoid harm that is not appropriately balanced by compensating medical benefits. Furthermore, if a doctor misrepresents their knowledge by recommending an intervention that they know will cause harm and not be beneficial, they have vitiated the trust placed in them by society and the patient to apply their expertise appropriately.^52^

While the patient's demand for a harmful ART intervention offers *one* reason for the doctor to proceed with the intervention, it is a weak one, and is insufficient to justify overriding the doctor's PRM. In this regard, it is important to remember that not acceding to a patient's autonomous wishes (a position we suppor here) is not the same as an unjustifiable *violation* of autonomy. As Oakley and Cocking explain:

While doctors who refuse to meet certain patient requests will probably restrict or fail to promote patient autonomy, they clearly need not be *violating* or failing to respect the autonomy of the patient whose request they refuse to meet. For violating and failing to respect a patient's autonomy involves interfering with their self‐determination in ways that are unjustifiable, and in refusing to act on a patient's autonomous request … or to be provided with costly but futile treatment, a doctor need not be restricting the patient's autonomy *unjustifiably*.^53^

In other words, if a doctor refuses a patient's demand for a harmful intervention, the patient's autonomy has *perhaps* not been promoted, but justifiably so.

This scenario also illustrates how there can be a *gap* in justification between acceding to patient‐led demand and offering an intervention where evidence suggests that it will not be beneficial and/or cause direct harm. Compelling moral reasons need to be provided to bridge this gap. The desire to promote autonomy, while laudable, does not, on its own, create a sufficiently robust bridge.

Thus far, we have shown how PRM can help to guide doctors' decision‐making when their professional opinions as to what is ‘best’ diverge from what a patient requests or demands. However, while some, like Rhodes, have offered hierarchies and privileging of goals such as ‘seeking trust and being worthy of it’,^54^ there is room for reasonable disagreement as to how the various goals of medicine and corresponding elements of PRM (whether these are professional standards, character traits or other principles) should be defined, specified and prioritised. PRM (like any form of principle‐based reasoning) cannot, therefore, be completely action‐guiding. Rather, it serves to highlight the relevant considerations that need to be specified and balanced and where arguments for a particular course of action (e.g., offering the double embryo transfer in Scenario 3) would need to be backed by especially strong justifications.

However, despite these limitations of PRM, we believe that PRM is not such a loose concept that it cannot offer parameters around what is acceptable. Furthermore, we believe that PRM can be particularly action‐guiding when it comes to clinical decision‐making in commercialised healthcare. We explore this in Section 5.

## 5 PROFESSIONAL ROLE MORALITY IN A COMMERCIAL SETTING

The analysis that we have provided so far discusses the issue of patient‐led demand in any health funding context. This issue may, however, be more important and more challenging in commercial settings. There are three reasons for this. First, while patients access information about many types of health interventions from peers, social media and Internet ‘research’, interventions that are offered in commercial settings are advertised more widely and intensively than those offered in publicly funded health systems. A patient seeking ART is likely to receive marketing messages about likely success and about ‘add‐ons’ from advertisements and from clinic websites.^55^ This, in turn, inevitably increases patient‐led demand for interventions. A second difference between publicly and privately funded healthcare is that, because doctors are competing with one another in private settings, they offer a wider range of interventions in order to differentiate themselves and respond to a wider range of patient/consumer preferences. This increases the likelihood that patients will hear about and ask for interventions. Third, commercialised settings are also settings in which doctors have financial interests, leading to explicit or implicit pressure on them to satisfy patients’ demands to prevent them from taking their ‘business’ elsewhere.^56^

While most forms of bioethical reasoning would likely support the view that commercial considerations should never take precedence over patient well‐being, PRM offers a compelling kind of argument for this view. This is because PRM is derived from a particular kind of social institution—a profession—that is, by definition, distinct from a marketplace.^57^ While markets, like professions, can contribute to human flourishing (by empowering consumers and maximising individual choice), it is broadly accepted that there is also an important place in society for social institutions that are driven and shaped by other considerations. Institutional theorists have articulated these differences in terms of ‘institutional logics’, which comprise shared patterns of beliefs, values and assumptions that prescribe particular ways of thinking and doing.^58^ In theory, a ‘market logic’ emphasises goods and practices such as voluntary exchange of resources, support for competition, private ownership and—of particular relevance to the issue of patient‐led demand—voluntary, informed and rational decision‐makers driven by self‐interest. A ‘professional logic’, in contrast, emphasises the pursuit and maintenance of specialised expertise and knowledge, professional standards and peer review and, crucially for patient‐led demand, responding to clients' needs (as opposed to just desires) and promoting their well‐being.^59^

This is not to say that commercial and professional institutions are entirely separate or that there is no room in professions for commercial transactions. It is also recognised that professionals working in commercial settings might need at times to adapt their practices so that they can remain financially viable (for example, an ART clinic might need to conduct a certain number of IVF cycles to offset other costs). But *responsible* management of the profit motive is a core part of PRM. For instance, if, instead of being guided by medical ideals, a doctor's guiding aim in practicing medicine is to enhance their clinic's commercial interests, they are, as Oakley and Cocking explain, being literally *mis*‐guided,^60^ or, as Cullity puts in in relation to implant surgery, they are ‘deliberating badly’:

In considering whether to recommend surgery to your patients, if you deliberate by weighing up their medical needs against the financial inducements offered by the implant manufacturer, you are deliberating badly, even if you conclude that the medical reasons prevail. It is wrong to think that the financial benefit to you is outweighed: it is medically irrelevant… [I]n presenting yourself as a doctor, you are assuring your patients that you will exercise a particular kind of agency—the agency of someone acting in the capacity of a doctor. On actions performed in that capacity, the only considerations that have a bearing as reasons are medical reasons … your professional role specifies a certain kind of agency, and the reasons bearing on the exercise of that kind of agency are restricted.^61^

This has important implications for responding to patient‐led demand for healthcare interventions in commercialised settings. While there is room in professional logics for responsiveness to clients', patients' or even consumers' wishes (as evident in the inclusion of principles such as ‘respect for autonomy’ in lists of PRM), the nature of a ‘profession’ means that autonomy has a specific meaning (more akin to respect for persons than consumer satisfaction), so when it is appealed to, this needs to be done with care. Instead of simply acceding to demands, PRM highlights the importance of doctors ensuring that patients’ requests or demands are truly informed and have not been distorted by misleading marketing practices. In practice, this would entail not simply taking a patient's request or demand for an intervention at face value, but rather ‘unpacking’ it to ensure that it is adequately informed and balanced by considerations that would not be evident in even technically ‘truthful’ advertising.

PRM also highlights the need for doctors to be alert to their own (conscious or unconscious) motivations and potential financial conflicts of interest. Because pressures are often implicit, effects on judgement are often unconscious, and commercial and non‐commercial reasons for acceding to patient‐led demands are often intertwined. PRM also requires that doctors adhere to professional guidelines generated by independent medical groups and subject their practice to regular peer review.

PRM might also suggest that ART doctors and their regulators have an obligation to address the broader cultural issues of commercial influences in ART provision. In this regard, it is noteworthy that clinics are often run by people who are *not* doctors. They also employ external marketing agencies to attract patients. Therefore, because a doctor's PRM entails being alert to the distorting effects of commercial influences on their medical decisions, there is arguably a role obligation (at least at the collective level, or with appropriate support) to help improve the industry. This, in turn, will make it easier for individual practitioners to fulfil their role obligations and achieve the goals of medicine.^62^

## 6 CONCLUSION

In this paper, we have argued that acceding to patient‐led demand is not necessarily incompatible with fulfilling the professional role obligations of a doctor, but, it might be so—particularly when patients demand interventions that lack evidence of safety or efficacy, or are known to be harmful. In these cases, acceding to a patient's demand needs strong moral justification in order for it to be compatible with the professional role obligations of a doctor. We have also argued that PRM reflects the workings of a type of social institution—a profession—that has an important place in promoting human flourishing via mechanisms that allow for commercial activity but are distinct from markets. This means that, in commercialised healthcare settings such as ART, doctors have an obligation to counter the perverse effects of market forces such as misleading advertising and conflicts of interest. Only then can doctors be said to be acting within the bounds of PRM.

## ETHICS STATEMENT

The qualitative interviews noted in this paper were conducted with research ethics approval from the University of Sydney. Approval number: 2020/320.

## Footnotes

1 Fauser, B. (2019). Towards the global coverage of a unified registry of IVF outcomes. *Reproductive BioMedicine Online*, *38*(2), 133–137.

2 While 33% of ART patients have a baby as a result of a single IVF cycle, and somewhere between 54% and 77% have a baby by their eighth treatment cycle, the chances of success decline with increasing age and additional cycles. See, for example, Chambers, G., Paul, R., Harris, H., Fitzgerald, O., Boothroyd, C., Rombauts, L., Chapman, M. G., & Jorm, L. (2017). Assisted reproductive technology in Australia and New Zealand: Cumulative live birth rates as measures of success. *Medical Journal of Australia*, 207(3), 114–118; Health Care Complaints Commissioner Victoria. (2020). *Inquiry into assisted reproductive treatment practices in Victoria*. Victoria. Retrieved June 17, 2022, from https://hcc.vic.gov.au/sites/default/files/media-document/hcc_inquiry_into_assisted_reproductive_treatment_practices_in_victoria_report__3.pdf

3 Add‐ons aim to achieve a variety of ends such as the selection of ‘better’ embryos (e.g., time‐lapse imaging), improving the chances of embryo implantation in the uterus (e.g., endometrial scratching, platelet‐rich plasma injection) or improving the likelihood of a pregnancy being sustained (e.g., immunosuppressant treatments). For debate over add‐ons, see, for example, Wilkinson, J., Malpas, P., Hammarberg, K., Mahoney Tsigdinos, P., Lensen, S., Jackson, E., Harper, J., & Mol, B. W. (2019). Do à la carte menus serve infertility patients? The ethics and regulation of in vitro fertility add‐ons. *Fertility and Sterility*, *112*(6), 973–977.

4 Australian Government Department of Health. (2020). *Medicare benefits schedule review taskforce: Taskforce report on gynaecology MBS Items*. Australian Government Department of Health; Chambers, G., Sullivan, E., & Ho, M. (2006). Assisted reproductive technology treatment costs of a live birth: An age‐stratified cost–outcome study of treatment in Australia. *Medical Journal of Australia*, 184(4), 155–158.

5 Elwyn, G., Laitner, S,. Coulter, A,. Walker, E,. Watson, P., & Thomson, R. (2010). Implementing shared decision making in the NHS. *BMJ*, *34*(7780), 971–973.

6 Childress, J. (2017). Needed: A more rigorous analysis of models of decision making and a richer account of respect for autonomy. *American Journal of Bioethics*, *17*(11), 52–54; Lupton, D. (1997). Consumerism, reflexivity and the medical encounter. *Social Science & Medicine*, *45*(3), 373–381.

7 See, for example, Lensen, S., Hammarberg, K., Polyakov, A., Wilkinson, J., Whyte, S., Peate, M., & Hickey, M. (2021). How common is add‐on use and how do patients decide whether to use them? a national survey of IVF patients. *Human Reproduction*, 36(7), 1854–1861.

8 We acknowledge complexity around concepts such as ‘best interests.’ We use these here in an indicative sense, to illustrate the kinds of considerations that may be relevant to determining when offering an intervention that a patient demands is prima facie appropriate. Other considerations, such as resource allocation, will also be relevant in determining the appropriateness of offering interventions.

9 In using the word ‘demand’, we are not suggesting that patients who ‘demand’ interventions are doing so in an aggressive or unreasonable way. We also note that a clear conceptual distinction between ‘patient‐led demand’ and related notions, such as being ‘patient centric’ in one's practice, may not always be possible. One practitioner may see a decision as being patient centric, while another may see the same decision as responding to patient‐led demand.

10 Latham, S., & Norwitz, E. (2009). Ethics and “cesarean delivery on maternal demand”. *Seminars in Perinatology, 33*, 405–409; 10; Vedam, S., Stoll, K., McRae, D., Korchinski, M., Valasquez, R., Wang, J., Patridge, S., McRae, L., Martin, R. E. Jolicoeur, G., & CCinBC Steering Committee. (2019). Patient‐led decision making: Measuring autonomy and respect in Canadian maternity care. *Patient Education and Counseling, 102*, 586–594.

11 Copur, M. (2019). Supporting data vs patient requests in oncology: When the two don't coincide. *Oncology, 33*, 156–158; Gogineni, K., Shuman, K., Chinn, D., Gabler, N., & Emanuel, E. (2015). Patient demands and requests for cancer tests and treatments. *JAMA Oncology, 1*, 33–39.

12 Coenen, S., Michiels, B., Renard, D., Denekens, J., & Royen, P. (2006). Antibiotic prescribing for acute cough: The effect of perceived patient demand. *British Journal of General Practice*, *56*, 183–190.

13 See, for example, Siegel‐Itzkovich, J. (2006). Patients demanding access to cancer drugs call off hunger strike. *BMJ: British Medical Journal, 332*(7554), 1352.

14 Chiodo, G., & Tolle, S. (1993). Requests for treatment: Ethical limits on cosmetic dentistry. *General Dentistry, 41*, 16–20; Chiodo, G., & Tolle, S. (2001). Cosmetic treatment, autonomy, and risks: ‘If you don't do it, I'll go to a dentist who will’. *General Dentistry, 49*, 16–18, 20, 22; Simonsen, R. (2007). Commerce versus care: Troubling trends in the ethics of esthetic dentistry. *Dental Clinics of North America, 51*, 281–287.

15 Blakely, B., Williams, J., Mayes, C., Kerridge, I., & Lipworth, W. (2019). Conflicts of interest in Australia's IVF industry: An empirical analysis and call for action. *Human Fertility*, *22*(4), 230–237; Iacoponi, O., van de Wiel, L., Wilkinson, J., & Harper, J. C. (2022). Passion, pressure and pragmatism: How fertility clinic medical directors view IVF add‐ons. *Reproductive BioMedicine Online*, *45*(1), 169–179. https://doi.org/10.1016/j.rbmo.2022.02.021

16 Supporting information containing the interview topics and empirical methodology for this project, is available from the corresponding author. Empirical results from this study are reported in separate publications, for example, Gallagher, S., Kerridge, I., Newson, A., Attinger, S., Norman, R., & Lipworth, W., (2023). Moral justification for the use of ‘add‐ons’ in assisted reproductive technology (ART): experts' views and experiences. *Reproductive Biomedicine Online*, published 21 October 2023. https://doi.org/10.1016/j.rbmo.2023.10637

17 Weimer, S. (2018). Evidence‐responsiveness and the ongoing autonomy of treatment preferences. *HEC Forum*, 30, 211–233.

18 Bullock, E. (2018). Paternalism and the practitioner/patient relationship. In K. Grill & J. Hanna (Eds.), *The Routledge handbook of the philosophy of paternalism* (pp. 311–322). Routledge.

19 Wasserstrom, R. (1984). Roles and morality. In D. Luban (Ed.), *The good lawyer: Lawyers’ roles and lawyers’ ethics* (pp. 25–37). Rowman & Allanheld.

20 Chappell, S. (2019). Roles and reasons. In T. Dare & C. Swanton (Eds.), *Perspectives in role ethics: Virtues, reasons, and obligation* (p. 194). Taylor & Francis Group.

21 Wolfendale, J. (2007). *Torture and the military profession*. Palgrave Macmillan UK. Also, note that there other accounts of professions, for example, as a ‘calling’ or ‘vocation’, or even as groups that emerge as expressions of power and self‐interest; See Friedson, E. (1989). Theory and the professions. *Indiana Law Journal*, 64, 423–433. We do not take these views of professions in this article.

22 While we recognise ongoing debate into questions such as whether human reproduction is central to flourishing, or whether social or medical infertility is a health condition, we set such debates to one side in this paper. We recognise that ART *is* provided, is publicly funded or cofunded in many countries, and is offered by doctors. That is, we are making our argument within the status quo. Inasmuch as reproduction is empirically seen as central to human flourishing for people (whether that is justified or not), then it is also acceptable to claim that an ART doctor is a professional role.

23 Wolfendale, op. cit. note 21, p. 9.

24 Camenisch, P. (1983). On being a professional, morally speaking. In B. Baumrin & B. Freedman (Ed.), *Moral responsibility and the professions* (pp. 42–61). Haven Publications.

25 Bayles, M. (1989). *Professional ethics* (2nd ed.). Wadsworth.

26 Hardimon, M. (1994). Role obligations. *Journal of Philosophy, 91*(7), 333–363.

27 Alexandra, A., & Miller, S. (2009). *Ethics in practice: Moral theory and the professions*. UNSW Press.

28 Oakley, J., & Cocking, D. (2001). *Virtue ethics and professional roles* (p. 89). Cambridge University Press.

29 While we do not have the scope to explore the connection in detail, this teleological structure of professional goals can also be a good example of how *virtue ethics* can be applied practically (Oakley & Cocking, op. cit. note 28; Oakley, J. (2018). Creating regulatory environments for practical wisdom and role virtues in medical practice. In D. Carr (Ed.), *Cultivating moral character and virtue in professional practice* (1st ed., pp. 83–89). Routledge). Virtue ethics has a focus on functions and ends in a similar way to professional practice's goal‐directed structure. A virtue, for instance, is just whatever allows something to perform its function. The function of a knife is to cut things, so a virtue of a knife is being sharp—it would not be a good knife if it lacked this virtue. Something counts as a virtue in medicine insofar as it helps the doctor to achieve the purpose of their profession. Using sound medical reasons to treat a patient, then, is plausibly a virtue because it helps to achieve the goal of acting in the best interests of the patient's health. In other words, seeking to uphold one's professional role obligations likely makes someone a virtuous doctor. Doctors are obligated to act from the virtues of their profession to achieve their goals, purposes and functions.

30 Miller, F., & Brody, H. (1995). Professional integrity and physician‐assisted death. *The Hastings Center Report, 25*(3), 8–17.

31 Hanson, M., & Callahan, D. (2000). The goals of medicine: Setting new priorities. In M. Hanson & D. Callahan (Eds.), *The goals of medicine* (pp. 1–54). Georgetown University Press.

32 Rhodes, R. (2020) *The trusted doctor: Medical ethics and professionalism*. Oxford University Press. Indeed, Rhodes notes that the obligations have been ‘Assimilated into the practice of medicine since at least the time of Hippocrates. They have been accepted by physicians as the duties that they must fulfil, and they are articulated in short and lengthy lists posted in the codes of ethics on the websites of medical societies’ (p. 47).

33 Miller & Brody, op. cit. note 30. p. 11.

34 Salloch S. (2016). Same same but different: Why we should care about the distinction between professionalism and ethics. *BMC Medical Ethics*, *17*, 1–6; Buyx, A. M., Maxwell, B., and Schöne‐Seifert, B. (2008). Challenges of educating for medical professionalism: Who should step up to the line? *Medical Education*, *42*(8), 758–764.

35 Dunn, M. (2016). On the relationship between medical ethics and medical professionalism. *Journal of Medical Ethics*, 42, 625–626. In ART, this would include issues such as the moral status of gametes and embryos, or the moral status of future persons.

36 Cruess, S., Johnston, S., & Cruess, R. (2002). Professionalism for medicine: Opportunities and obligations. *Medical Journal of Australia*, *177*(4), 208–211.

37 Backof, J., & Martin, C. (1991). Historical perspectives: Development of the codes of ethics in the legal, medical and accounting professions. *Journal of Business Ethics, 10*(2), 99–110.

38 Parker, M. (2010). Normative lessons: Codes of conduct, self‐regulation and the law. *Medical Journal of Australia*, *192*(11), 658–660.

39 ABIM Foundation. (2004). Medical professionalism in the new millenium: A physicians' charter. https://abimfoundation.org/what-we-do/physician-charter

40 Pellegrino, E. (2000). The goals and ends of medicine: How are they to be defined? In M. Hanson & D. Callahan (Eds.), *The goals of medicine* (pp. 55–68). Georgetown University Press.

41 For example, see Oakley & Cocking, op. cit. note 28; Cullity, G. (2019). Deliberative restriction and professional roles. In T. Dare, & C. Swanton (Eds.), *Perspectives in role ethics: Virtues, reasons, and obligation* (pp. 173–193), Taylor & Francis Group; Alexandra & Miller, op. cit. note 27.

42 Brody, H., & Miller, F. (1998). The internal morality of medicine: Explication and application to managed care. *The Journal of Medicine and Philosophy*, *23*(4), 384–410.

43 Miller, F., Brody, H., & Chung, K. (2000). Cosmetic surgery and the internal morality of medicine. *Cambridge Quarterly of Healthcare Ethics, 9*(3), 353–364.

44 Wicclair, M. (2008). Is conscientious objection incompatible with a physician's professional obligations? *Theoretical Medicine and Bioethics, 29*(3), 171–185.

45 There is likely no clear line between each scenario, but rather a spectrum. Also, while our focus is on add‐ons in the three scenarios, a similar line of reasoning will apply to other contentious areas where patient‐led demand plays a role, for example, offering additional IVF cycles where these are unlikely to succeed. Some scenarios are likely to be consistent with PRM, and others more morally problematic, dependent on evidence levels and risk to patients.

46 We draw on harm and evidence in particular because participants in our research raised these issues. See also op. cit. note 16.

47 Hanson & Callahan, op. cit. note 31.

48 Wrigley, A., Watts, G., Lipworth, W., & Newson, A.J. (2023). Hope and exploitation in commercial provision of assisted reproductive technologies. *Hastings Center Report*, *53*(5), 30‐41. https://doi.org/10.1002/hast.1513

49 We note that if this technique is used for other indications, such as timing of embryo transfer, its utility decreases. See, for example, Awonuga, A. O., Camp, O. G., Abu‐Soud, H. M., Rappolee, D. A., Puscheck, E. E., & Diamond, M. P. (2023) Determinants of embryo implantation: Roles of the endometrium and embryo in implantation success. *Reproductive Sciences*, *30*, 2339–2348.

50 We recognise that there could be complexity surrounding the ‘nonidentity problem’ when talking about any harm to offspring. However, it is beyond our scope, and not the focus of this paper, to address this problem. For more on the non‐identity problem and ART, see Roy, M. C., Dupras, C., & Ravitsky, V. (2017). The epigenetic effects of assisted reproductive technologies: Ethical considerations. *Journal of the Developmental Origins of Health and Disease, 8*(4), 436–442.

51 Raz, J. (1986). *The morality of freedom*. Oxford; Cullity, op. cit. note 41.

52 Miller & Brody, op. cit. note 30; Rhodes, op. cit. note 32.

53 Oakley & Cocking, op. cit. note 28, p. 85 (emphasis in original).

54 Rhodes, op. cit. note 32.

55 van de Wiel, L., Wilkinson, J., Athanasiou, P., & Harper, J. (2020). The prevalence, promotion and pricing of three IVF add‐ons on fertility clinic websites. *Reproductive BioMedicine Online, 41*(5), 801–806.

56 As noted in Section 2, this was prevalent in our empirical data. See also op. cit. note 16.

57 Friedland, R., & Alford, R. (1991). Bringing society back in: Symbols, practices, and institutional contradictions. In W. Powell & P. Di Maggio (Eds.), *The new institutionalism in organizational analysis* (pp. 232–266). University of Chicago Press; Scott, W. R., Ruef, M., Mendel, P., & Caronna, C. (2000). *Institutional change and healthcare organizations: From professional dominance to managed care*. University of Chicago Press; Thornton, P., & Ocasio, W. (2008). Institutional logics. In R. Greenwood, C. Oliver, K. Sahlin & R. Suddaby (Eds.), *Handbook of organizational institutionalism* (pp. 99–129). Sage.

58 Thornton P., Ocasio W., & Lounsbury M. (2012). *The institutional logics perspective: A new approach to culture, structure and process*. Oxford University Press.

59 Scott, W. R., et al., op. cit. note 57.

60 Oakley & Cocking, op. cit. note 28, p. 101.

61 Cullity, op. cit. note 41, p. 179.

62 Unpacking the details of this responsibility is beyond the scope of this paper. Our normative aim has been to evaluate a doctor's best practice and thus show a justificatory gap between the best practice and how ART provision currently operates.

